# Identification of risk variants related to malignant tumors in children with birth defects by whole genome sequencing

**DOI:** 10.1186/s40364-022-00431-y

**Published:** 2022-11-16

**Authors:** Yichuan Liu, Hui-Qi Qu, Xiao Chang, Frank D Mentch, Haijun Qiu, Kenny Nguyen, Xiang Wang, Amir Hossein Saeidian, Deborah Watson, Joseph Glessner, Hakon Hakonarson

**Affiliations:** 1 Center for Applied Genomics (CAG), Children’s Hospital of Philadelphia, 3615 Civic Center Blvd, Abramson Building, PA 19104 Philadelphia, USA; 2 Department of Pediatrics, The Perelman School of Medicine, University of Pennsylvania, 19104 Philadelphia, PA USA; 3 Division of Human Genetics, Children’s Hospital of Philadelphia, 19104 Philadelphia, PA USA; 4 Division of Pulmonary Medicine, Children’s Hospital of Philadelphia, 19104 Philadelphia, PA USA; 5 Faculty of Medicine, University of Iceland, Reykjavik, Iceland

**Keywords:** Birth defect, Pediatric cancer, Whole genome sequencing

## Abstract

**Background:**

Children with birth defects (BD) are more likely to develop cancer and the increased risk of cancer persists into adulthood. Prior population-based assessments have demonstrated that even non-chromosomal BDs are associated with at least two-fold increase of cancer risk. Identification of variants that are associated with malignant tumor in BD patients without chromosomal anomalies may improve our understanding of the underlying molecular mechanisms and provide clues for early cancer detection in children with BD.

**Methods:**

In this study, whole genome sequencing (WGS) data of blood-derived DNA for 1653 individuals without chromosomal anomalies were acquired from the Kids First Data Resource Center (DRC), including 541 BD probands with at least one type of malignant tumors, 767 BD probands without malignant tumor, and 345 healthy family members who are the parents or siblings of the probands. Recurrent variants exclusively seen in cancer patients were selected and mapped to their corresponding genomic regions. The targeted genes/non-coding RNAs were further reduced using random forest and forward feature selection (ffs) models.

**Results:**

The filtered genes/non-coding RNAs, including variants in non-coding areas, showed enrichment in cancer-related pathways. To further support the validity of these variants, blood WGS data of additional 40 independent BD probands, including 25 patients with at least one type of cancers from unrelated projects, were acquired. The counts of variants of interest identified in the Kid First data showed clear deviation in the validation dataset between BD patients with cancer and without cancer. Furthermore, a deep learning model was built to assess the predictive abilities in the 40 patients using variants of interest identified in the Kids First cohort as feature vectors. The accuracies are ~ 75%, with the noteworthy observation that variants mapped to non-coding regions provided the highest accuracy (31 out of 40 patients were labeled correctly).

**Conclusion:**

We present for the first time a panorama of genetic variants that are associated with cancers in non-chromosomal BD patients, implying that our approach may potentially serve for the early detection of malignant tumors in patients with BD.

**Supplementary Information:**

The online version contains supplementary material available at 10.1186/s40364-022-00431-y.

## Background

Birth defects (BD) affect 1 in every 33 babies (~ 3%) born in the United States each year based on the statistics by the Centers for Disease Control and Prevention (CDC, https://www.cdc.gov/ncbddd/birthdefects/index.html ). BDs contribute to long-term disability, which takes a significant toll on affected individuals, families, health care systems and societies world-widely (https://www.who.int/news-room/fact-sheets/detail/birth-defects ). Risk of cancer is significantly increased in BD patients with either non-chromosomal or chromosomal anomalies [1]. Based on a population-based assessment of 10 million live births, children with chromosomal anomalies are over 11 times more likely to be diagnosed with cancer compared with children without any BD, and the likelihood for children with non-chromosomal BD increases 2.5-fold [2]. Accordingly, the cancer risk in BD children with non-chromosomal anomalies cannot be neglected considering the large effect size.

The co-occurrence of BD in patients with pediatric cancer may share common nexus in the gene pathways governing signal transduction and cell growth, e.g., the Wnt signaling pathway [3]. Unfortunately, few pathways or corresponding genes/variants have been identified in this context due to limited research efforts. Based on the multi-hit theory of childhood cancer [4], the efforts in this study focused on identifying genomic mutations, in both coding and non-coding regions, which are enriched in BD patients with cancers compared to BD patients without cancers based on the Whole Genome Sequencing (WGS) analysis. In this regard, we assembled one of the largest pediatric oncology and birth defect projects in children, as a part of the Gabriella Miller Kids First program project (https://kidsfirstdrc.org/). The major objective was to identify the functional molecular pathways and evaluate the performance of risk prediction with genomic information based on the mutations identified.

## Methods

### Discovery and validation cohorts

**Fig. 1:**
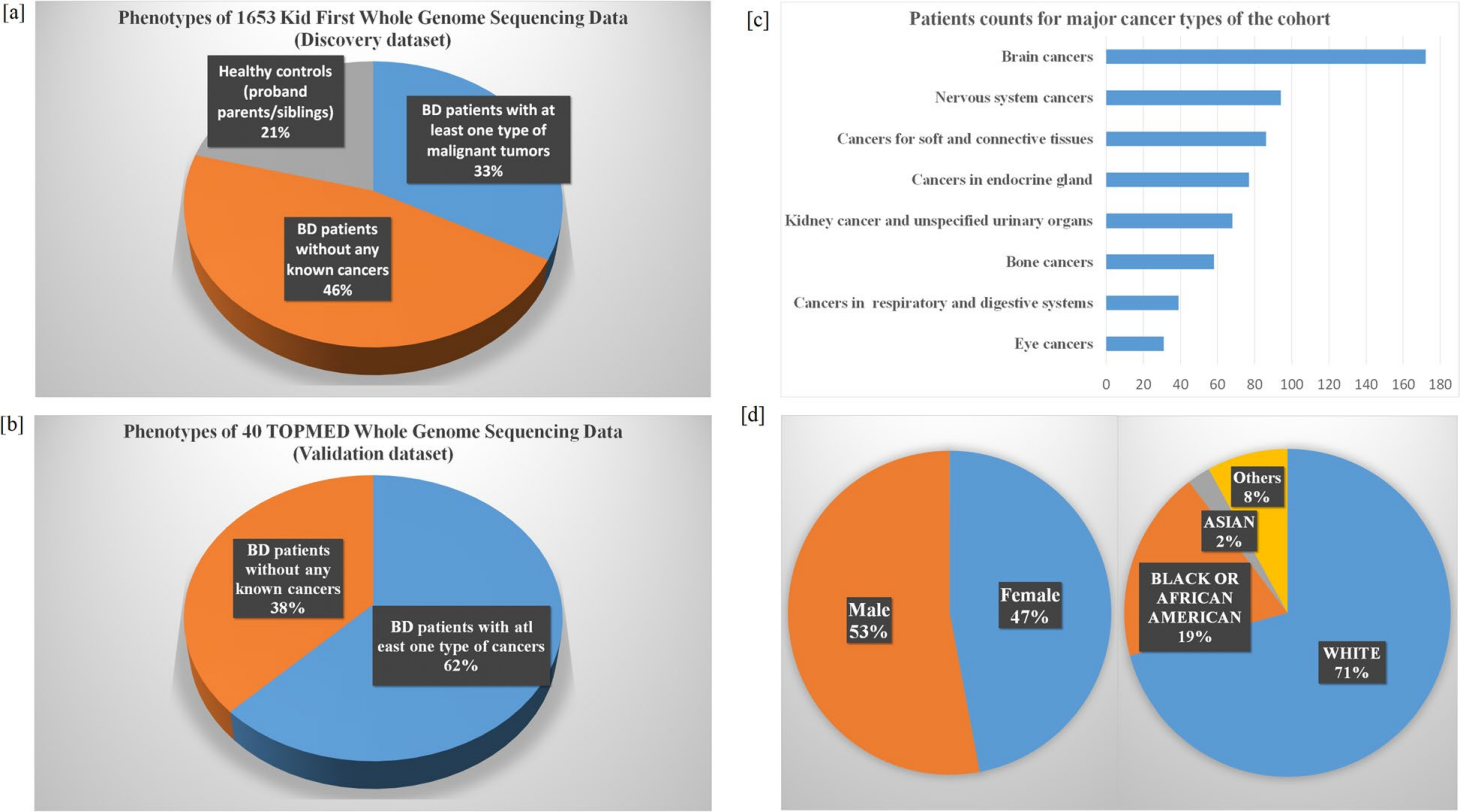
Demographic information of the cohort. **a** Phenotype information of the discovery cohort; **c** Phenotype information of the validation cohort; **c** Number of patients with cancers; **d** Gender and ethnicity distribution of the cohort

The patients with or without co-occurrence of pediatric onset cancers were selected from the Center for Applied Genomics (CAG) Biorepository for birth defects (BD). The BD and cancer diagnosis were based on the International Classification of Diseases (ICD) codes ICD-9 and ICD-10. All the CAG patients were recruited at the Children’s Hospital of Philadelphia (CHOP) and coupled to Electronic medical records (EMRs) of CHOP established in 2003. Altogether, 1308 probands with non-chromosomal anomalies were studied, including 541 BD patients with at least one type of malignant tumors and 767 BD patients without any known cancers. In addition, 345 healthy controls without BD or cancer who are parents/siblings of the probands were also investigated in comparison (Fig. 1a). The patients’ counts for malignant cancers and demographic information including gender and ethnicities were shown in Fig. 1c and d.

The patients in the validation dataset were also recruited by CAG at CHOP, including research participants who had undergone WGS through the NHLBI Trans Omics for Precision Medicine (TOPMed) Program (https://www.ncbi.nlm.nih.gov/projects/gap/cgi-bin/study.cgi?study_id=phs001661.v2.p1) in a different project. In total, 40 BD patients were identified in the CAG biobank, including 25 with at least one type of cancer diagnosis (Fig. 1b).

All the patients were recruited during regular hospital visits at multiple clinics, including the emergency room, ambulatory settings or surgical settings, through the general pediatric clinics or CHOP’s specialty pediatric practices. The patients were in the age range of 0–21 years and receiving health care at CHOP. Parental consent was obtained for individuals under 18 years of age and assent was also obtained for subjects aged 7–17 years. The consent allows samples to be obtained and analyzed using the genomic technologies included herein, to address the research questions proposed.

### Electronic medical record (EMR) and electronic health record (EHR) data

CAG at CHOP maintains a de-identified extract of clinical data from the CHOP EMR and EHR databases of consented patients. This database contains longitudinal information about visits, diagnoses, medical history, prescriptions, procedures, and lab tests with all information coded and de-identified. For this study the health status of de-identified individuals was classified based on the International Classification of Diseases (ICD) codes (ICD-9 and ICD-10) associated with clinical visits and entered in the medical health record.

### Processing and variant detection by WGS

Whole genome sequencing (WGS) was done at 30X coverage for the 1653 individuals from the Gabriella Miller Kids First project, by the genomics platform of the Broad Institute. The variant call format (VCF) files of WGS were generated using the Illumina DRAGEN (Dynamic Read Analysis for GENomics) Bio-IT Platform (Illumina, San Diego, CA), aligned to the GRCh38/hg38 human genome assembly. For the validation dataset, WGS VCF files of participating individuals were extracted from the TOPMED database directly. The annotations for the variants were generated using the ANNOVAR software developed by our group [5], and the variants were further divided into three groups based on their genomic locations, i.e., variants in coding regions, variants in non-coding variants including intronic variants, variants in untranslated regions (UTR) or in non-coding RNAs, and variants in intergenic regions.

### Genetic variants correlated with malignant tumors in BD patients

A variant is considered as “same” or “identical” if the mutation is at the same genomic locus with the same alternative allele. Variants that occur in at least three individuals in the 541 BD patients with malignant tumors, and were absent in the 767 BD-only patients and the 345 healthy family controls, were identified as recurrent variants of interest. Selected variants were then mapped to the corresponding genes/non-coding RNAs based on their genomic location. The number of the variants of interest in BD patients with malignant tumors were considered as the “weight” for each corresponding gene/non-coding RNA.

Multiple machine learning algorithms including random forest and forward feature selection (ffs) were applied to further select the informative gene/non-coding RNA features. Random forest is one of the most widely used algorithms for feature selection, which computes relative importance or contribution of each gene feature in the prediction model, then scales the relevance down so that the sum of all scores is 1. All the genes/non-coding RNAs with relative importance less than 1E-5 were removed. The second algorithm ffs is one of the most common methods to reduce number of features for machine learning inputs by trying to find the best features which improve the performance of the prediction model. The modeling codes were based on the Scikit-learn package in Python language [6] and functional enrichment analysis was performed using WebGestalt (WEB-based Gene SeT AnaLysis Toolkit) [7].

### Validation of selected variants using the independent dataset

Variants of interest in genes/non-coding RNAs identified in the discovery cohort were examined in the validation dataset, and mutation load boxplots of BD patients with cancers (group A) versus BD patients without cancers (group B) were generated. These included variants in coding regions, intronic regions, untranslated regions (UTR), non-coding RNA regions, and intergenic regions, respectively.

Multi-layer perceptron (MLP) from the Scikit-learn package (version 0.21.3) [6] was applied as the deep learning model based on multiple different types of coding/non-coding variants. Parameters for deep learning model, including maximum iterations, alpha value in L2 regularization, activation functions, solvers, learning rate, number of layers, and numbers of neurons per layer, were optimized using ‘gp_minimize’ function from the scikit-optimise 0.7.2 python library. The prediction has been made for each patient to label either BD patient with cancer or BD patient without cancer.

## Results

### Functional enrichment of genes with selected variants

**Fig. 2:**
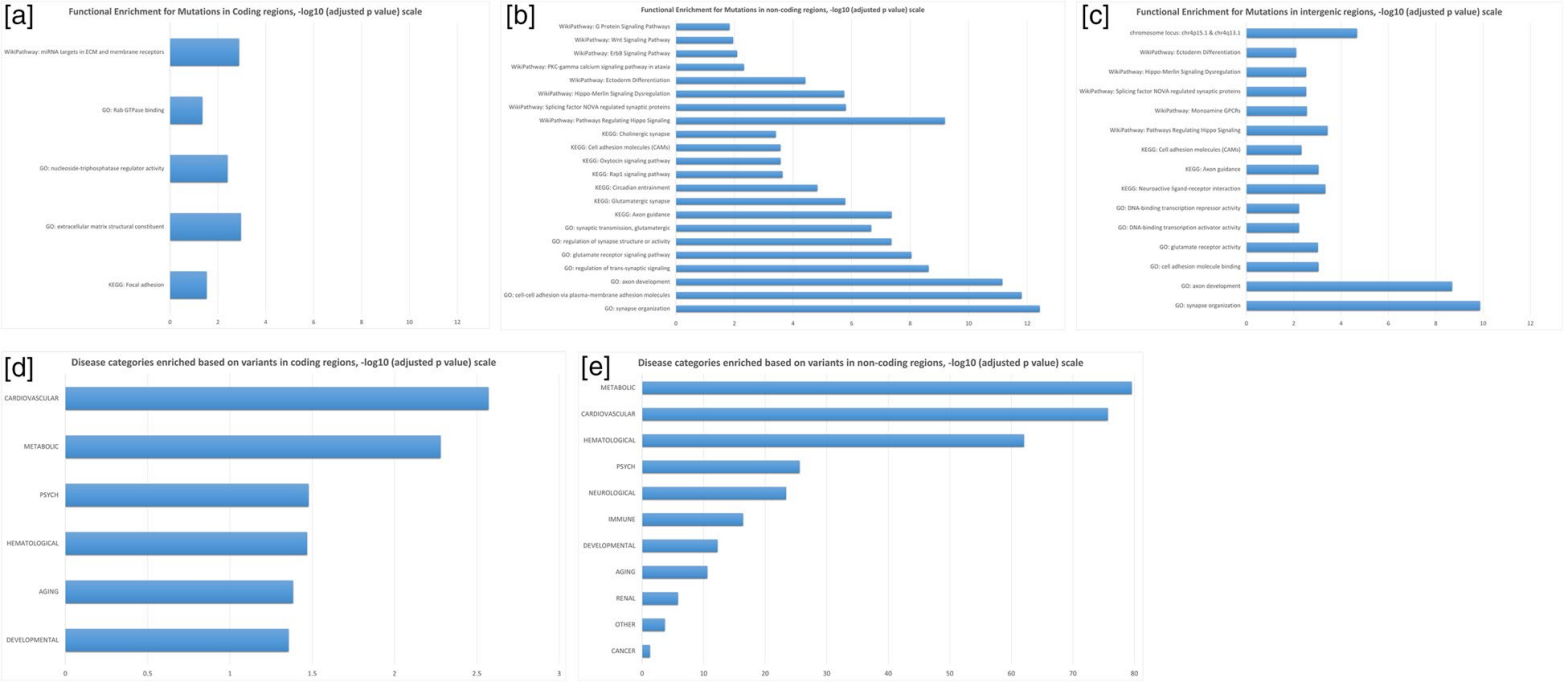
Functional pathway enrichment of genes in -log10 (adjusted p value) scale. **a** Genes with variants mapped to coding regions; **b** Genes with variants mapped to non-coding regions, including intronic variants, variants in untranslated region (UTR) or in non-coding RNAs; **c** Genes with variants mapped to intergenic regions Disease category enrichment of genes in -log10 (adjusted p value) scale. **d** Genes with variants mapped to coding regions; **e** Genes with variants mapped to non-coding regions

A total of 158,493 variants were selected as variants of interest based on the criteria described in the method section. Variants in coding regions were mapped to 611 genes, variants in non-coding regions were mapped to 1,829 genes/non-coding RNAs, and variants in intergenic regions were mapped to 1,719 genes/non-coding RNAs after filtering by machine learning models (Supplementary Table 2). The identified genes/non-coding RNAs were further analyzed using multiple functional enrichment algorithms including Gene Ontology (GO) (9), Kyoto Encyclopedia of Genes and Genomes (KEGG) (10), Wiki Pathways (11), Gene-Disease Associations Dataset (GAD)[8], and BDdb: a comprehensive platform for exploration and utilization of birth defect multi-omics data [9]. Multiple molecular function categories were identified with significantly statistical p values based on genes/non-coding RNAs corresponding to genomic variants in non-coding areas such as intronic, untranslated region (UTR), non-coding RNAs, and intergenic regions. Several oncology-based functional pathways showed significance of adjusted p values of less than 0.05 (Fig. 2). Genes containing multiple selected variants, including variants in coding/non-coding regions, were listed in Supplementary Table 3.

### Mutation load in the validation dataset

**Fig. 3:**
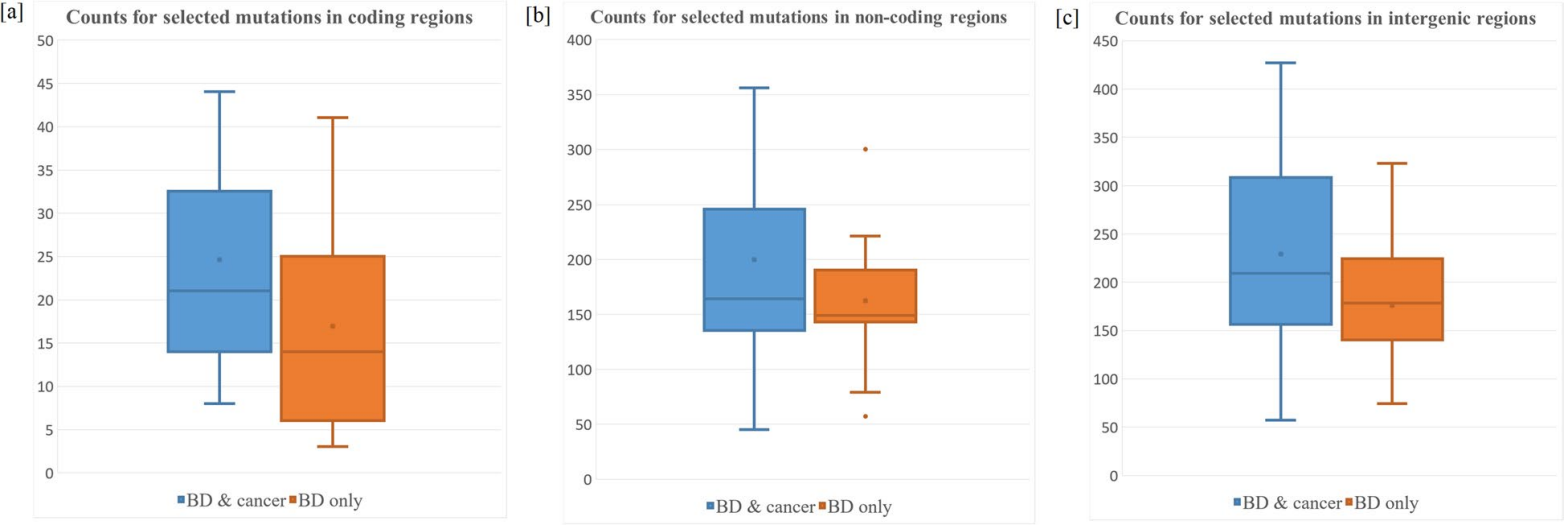
Boxplot of variant numbers between BD patients with cancers versus BD patients without cancers

The number of variants of interest was counted for each individual in the validation dataset, including 25 BD patients with cancer. While the selected variants are exclusively seen in BD patients with cancer in the Kid First discovery set, as expected, similar patterns of mutation loads were seen in the validation dataset. The boxplots (Fig. 3) shows that the counts of the selected variants are higher in BD patients with cancers compare to patients with only BD, indicating that the variants of interest have the potential to serve as biomarkers for the risk prediction of cancer in BD patients.

### Labeling accuracy in the validation cohort

**Fig. 4:**
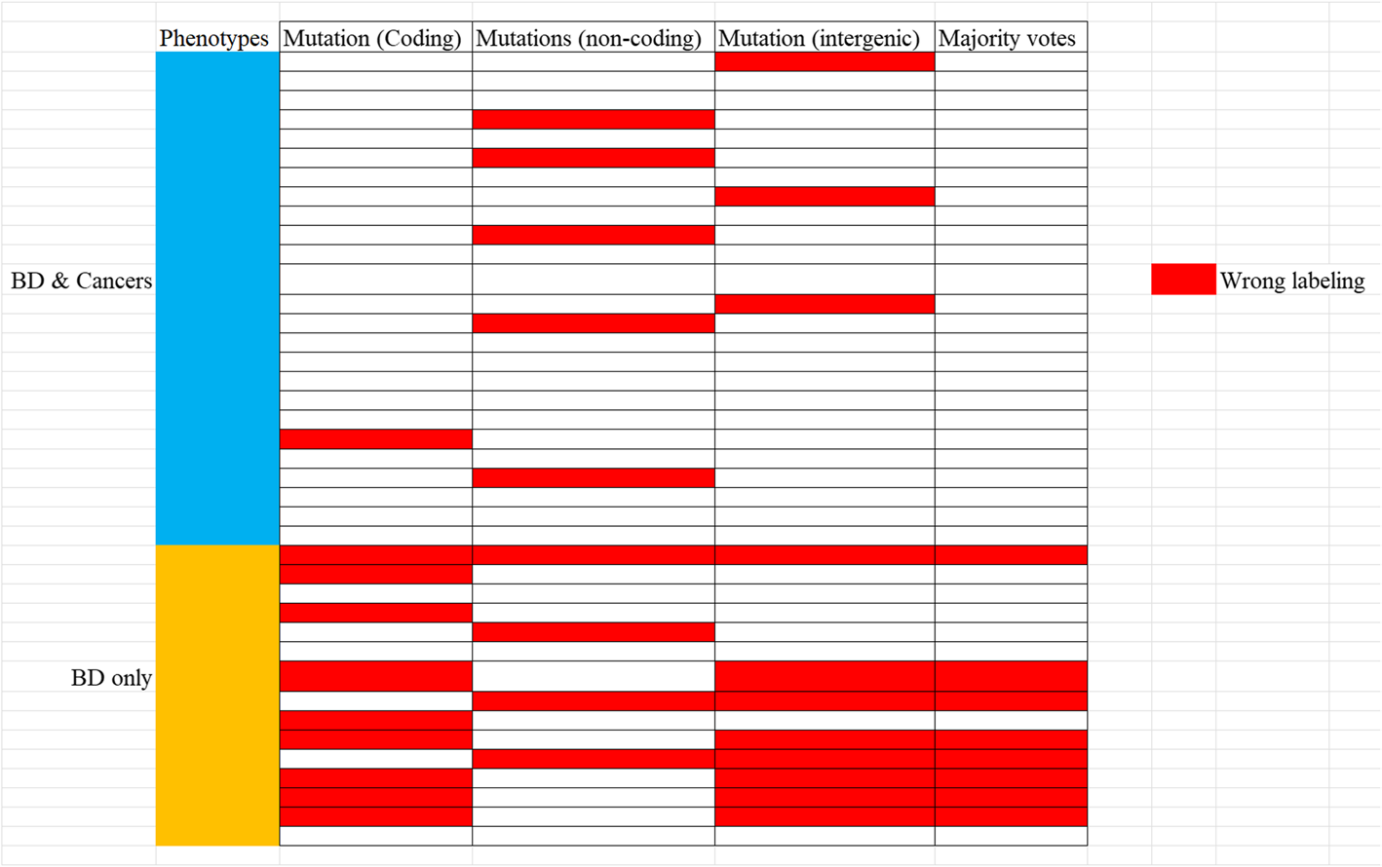
Labeling accuracies of validation dataset, with 40 independent patients from different projects

To generate quantitative measures of the biomarkers, we built a deep learning model to test the labeling accuracies as described in the method section. In the validation cohort, we assessed whether BD patients with/without cancers could be labeled correctly based on the identified genes/non-coding RNAs using variant counts as the weight factor. As shown in this study, labelling accuracies are similar for variants in coding regions (30 out of 40 labeling correctly), non-coding regions (31 out of 40 labeling correctly), and intergenic regions (29 out of 40 labeling correctly), whereas the missed targets are inconsistent (Fig. 4). An interesting finding is that if we combine the prediction results using the majority vote algorithm, the accuracy increased to 80% (32 out of 40 labeling correctly) and all the mis-labeling are BD patients without cancer.

## Discussion

Studies show that children with non-chromosomal birth defects (BD) are at least 2.5 times more likely to be diagnosed with cancer before 18 years of age, and the risk increases if more severe BDs are identified [2]. While this is of concern conceptually, it should be noted that the absolute risk remains low (< 1%) [2, 10], which means that biomarkers that are associate with malignant tumors in BD patients are likely to be important for the evaluation of cancer risk. This study is the first to evaluate variants of interest in BD patients with malignant tumors in comparison with BD patients without any cancers, based on 1,653 individuals with WGS data from the KidFirst DRC. The primary goal of this study is to explore the molecular functions of candidate variants for potential disease-causing effects and provide new information of how well these biomarkers may be applied to capture cancer risk as tested in the independent validation sample.

To select the informative variants from blood DNA samples, we required the variants to be exclusively occurring in BD patients with malignant tumors, and BD patients without any cancers having none. There are several potential issues that need to be addressed. First is the false positive hits, such as isolated variants identified in cancer patients by chance alone. To address this issue, we required the variants must occur in at least three unrelated cancer patients, and with none existing in any of the 345 family-based control subjects, who are healthy parents/siblings of the BD probands. The second issue is that acquired variation secondary to treatment couldn’t be differentiated in this study while we had limited access to patients’ treatment information. To help with this issue, we searched all variants of interests identified in this study through the Catalogue of Somatic Mutations In Cancer (COSMIC) database [11], no overlapped variant was identified. Since acquired somatic variants are mosaic, they might be removed in the QC processes of sequencing data. Therefore, it is reasonable to assume variants of interest in the study as causal variants. The last issue is overfitting due to the magnitude differences of DNA variants and experimental samples. To reduce the number of variants significantly while keeping the informative signals, selected variants of interest were first mapped to corresponding genes/non-coding RNA genomic regions, while the count of the variants serves as the weight in the corresponding gene/non-coding RNA. Consequently, two layers of filters (random forest plus forward feature selection) were applied to further select the most informative signals based on the weight, which is the mutation load. These steps reduced the gene/non-coding RNAs signals to acceptable size compared to data sample magnitude (611 genes for variants in coding regions; 1,829 genes/non-coding RNAs for variants in non-coding regions; and 1,719 genes/non-coding RNAs for variants in non-coding regions). The variant number was also reduced by ~ 30%, from 158,493 to 116,554.

Multiple cancer related networks/pathways were identified using functional enrichment analysis for the corresponding genes/non-coding RNAs containing variants of interest (Fig. 2a-c). For variants identified in coding regions, focal adhesion kinases (FDR = 0.029) are important mediators of growth-factor signaling in cell proliferation, survival, and migration[12], and have been considered as important therapeutic targets for immunotherapy in cancer [13, 14]. The miRNA targets in extracellular matrix (ECM) and membrane receptors (FDR = 0.0013) are believed to mediate migration, invasion, and metastasis of cancer cells[15]. For variants in non-coding genomic regions, such as intronic, untranslated region (UTR), and non-coding RNA regions, multiple pathways have been identified including Glutamatergic synapse (FDR = 1.66E-6), Circadian entrainment (FDR = 1.48E-5), Rap1 signaling pathway (FDR = 2.27E-4), Oxytocin signaling pathway (FDR = 2.67E-4), and ErbB Signaling Pathway (FDR = 0.008). Glutamatergic synapse is related to the progression and excitotoxicity of glioblastomas [16]. Circadian rhythm plays a key role in maintaining homeostasis of multiple physiological processes, and its disruption promotes tumorigenesis; therefore, it has been considered as a new target for cancer prevention and treatments [17]. Rap1 signaling regulates cell invasion/metastasis [18] and central nervous system (CNS) neuropeptide hormone oxytocin isa cancer biomarker found to link to multiple type of cancers through interactions with a number of factors in the microenvironment [19]. Multiple pathways enriched for intergenic variants corresponding genes/non-coding RNAs are overlapping with the enriched pathways for genes/non-coding RNAs with variants in intronic, untranslated region (UTR), and non-coding RNA regions, including Axon guidance (FDR = 9.07E-4 & 4.31E-8, respectively), Cell adhesion (FDR = 4.76E-3 & 2.69E-4), Pathways Regulating Hippo Signaling (FDR = 3.74E-4 & 6.52E-10), Splicing factor NOVA regulated synaptic proteins (FDR = 0.003 & 1.56E-6), Hippo-Merlin Signaling Dysregulation (FDR = 0.003 & 1.78E-6), and Ectoderm Differentiation (FDR = 0.008 & 3.8E-5). Genetic variants and observed altered expression of the Hippo pathway have the unique capacity to lead to tumorigenesis and thereby, promote the migration, invasion, and malignancy of cancer cells through cell cycle regulation [20, 21]. NOVA factors promote cell proliferation, colony formation, migration, and invasion by interacting with miRNA in cancer cells [22]. It is worth to mention that genes/non-coding RNAs with non-coding region variants present in a number of functionally enriched pathways/gene-sets with statistical significance (Fig. 2), indicating variants in non-coding genomic regions may play important roles in pediatric cancers in birth defect patients. Meanwhile, multiple disease categories, especially those related to birth defects, were significantly enriched (e.g., metabolic diseases, cardiovascular diseases, psychiatric diseases) (Fig. 2d and e).

There are 119 genes with at least two variants in coding regions and 478 genes with at least 20 variants in their non-coding regions (Supplementary Table 3). Genes with multiple coding variants are enriched in diseases associated with glycosylation, including Termination of O-glycan biosynthesis (FDR = 0.00012), O-glycosylation of proteins (FDR = 0.00077), and O-linked glycosylation (FDR = 0.012). Congenital disorders of glycosylation (CDG) is an umbrella term for a rapidly expanding group of over 130 rare genetic, metabolic disorders, associated with birth defects and cancers. For genes with multiple identified non-coding variants, they are enriched in biological pathways related to neural systems, including glutamatergic synapse (FDR = 0.0053) and axon guidance (FDR = 0.0053); cardiovascular system (cardiomyopathy, FDR = 0.0053); and pathways related to tumorigenesis, such as phospholipase D signaling pathway (FDR = 0.0053), ErbB signaling pathway (FDR = 0.033) and Cadherin signaling pathway (FDR = 0.015). In addition, enrichment of gene-set is identified in Autistic Disorder (FDR = 2.8e-10), Chagas Cardiomyopathy (FDR = 1.04e-11), as well as multiple types of cancers based on the OMIM database.

Six hundred eleven genes with coding variant were searched by the GAD and BDdb databases to identify previously reported associations with birth defects and cancers. Five genes, *AXIN2, BMP1, CR1, ERBB2*, and *RYR1*, have been recorded of association with birth defects and cancer (Table 1). *AXIN2* was reported of association with cleft lip [23] and mutations of this genes are associated with multiple types of cancers; mutations of *BMP1* were reported in patients with gastroschisis [24] and may serve as a therapeutic biomarker for gastric cancer patients [25]; *CR1* is related to tetralogy of Fallot [26], a type of congenital heart defects, associated with susceptibility to gallbladder cancer, and a prognosis predictor in non-small cell lung cancer [27]; *ERBB2*, a well-studied biomarker in breast invasive ductal carcinoma, lung adenocarcinoma, colon adenocarcinoma, bladder urothelial carcinoma, and invasive breast carcinoma, is also related to neural tube defect [28]; *RYR1* is a major contributor to nonsyndromic Tetralogy of Fallot [29], and higher expression level of *RYR1* in tumor is associated with advanced stage of the uterine serous cancer [30].

**Table 1:** Coding variants mapped genes known to associate with both birth defects and cancers

Gene ID	Associated Birth defects	Associated Cancers
AXIN2	Cleft Lip	Mutations in AXIN2 gene were found in patients with colorectal or hepatocellular carcinoma, prostate cancer, ovarian or lung cancer
BMP1	Gastroschisis	Therapeutic biomarker for gastric cancer
CR1	Tetralogy of Fallot	Susceptibility to gallbladder cancer and a prognosis predictor in non-small cell lung cancer
ERBB2	Neural tube defect	biomarkers in breast invasive ductal carcinoma, lung adenocarcinoma, colon adenocarcinoma, bladder urothelial carcinoma, and invasive breast carcinoma
RYR1	Tetralogy of Fallot	Prognostic biomarker of uterine cancer

We validated the identified variants by evaluating their prediction ability in an independent cohort. We selected 40 BD patients from an independent project, including 25 patients diagnosed with at least one type of cancer. The number of variants of interest were calculated for each patient. If the selected variants of interest from the 1,653 subjects in the discovery cohort represent the biological/medical characters of pediatric cancers in BD patients, similar patterns would be expected in the independent validation cohort. The boxplots in the validation cohort (Fig. 3) show clearly larger numbers of selected variants in BD patients with cancers compared to BD patients without cancers, for both variants in coding regions and those in non-coding regions. A deep learning model was further applied to assess the predictive abilities of the biomarkers. The labelling accuracies were stable at around 75% (30 out of 40 patients labeling correctly), and the mis-labeling individuals were inconsistent when using different feature factors in three different types of genomic regions (Fig. 4). Notably, combining labeling results using majority vote algorithms increased the accuracy to 80% with all eight mis-labeling patients belonging to the BD patients without cancer, leaving the overall accuracy in predicting this particular phenotype to random guess (7 out of 15 labelled correctly). The potential reasons may be due to genetic heterogeneity or incomplete penetrance underlying the molecular mechanisms of birth defects without cancer. In this regard, birth defects without cancer may be more difficult to be identified and captured comprehensively due to fewer phenotype terms (i.e. without cancers which have higher predictability).

## Conclusion

In conclusion, we conducted the first study to identify genetic variants of interest related to malignant tumors in patients with coexisting BD, leveraging one of the largest pediatric BD and oncology resources assembled. The results were validated in an independent cohort study of unrelated patients. Further research of the variants of interest identified in this study could unveil new insights into the underlying molecular mechanisms of BD with cancer and may offer new insight into optimal use of biomarkers in predicting and diagnosing cancer in BD patients at early stage.

## Supplementary Information

**Additional file** **1.**

**Additional file 2.**

**Additional file 3.**

## Data Availability

The KidFirst data could be accessed at Kids First Data Resource Portal (DRC) (https://portal.kidsfirstdrc.org/login). The TOPMED data has been uploaded to the database of Genotypes and Phenotypes (dbGaP, https://www.ncbi.nlm.nih.gov/gap/) with the accession number phs001661.v2.p1.
